# Online quantitative partial discharge monitor based on interferometry

**DOI:** 10.1038/s41598-020-76134-x

**Published:** 2020-11-04

**Authors:** Liang Xue, Yueyue Zhu, Chuankai Yang, Sisil Kumarawadu

**Affiliations:** 1 College of Electronics and Information Engineering, Shanghai University of Electric Power, Shanghai, 200090 People’s Republic of China; 2 State Grid Shaanxi Electric Power Research Institute, Hangtian Mid RD.669, Changan Dist, Xi’an, Shaanxi People’s Republic of China; 3 Department of Electrical Engineering, University of Moratuwa, Katubedda, Moratuwa, Sri Lanka

**Keywords:** Imaging and sensing, Electrical and electronic engineering

## Abstract

Interferometry-based online partial discharge (PD) monitor presented in this paper can detect the occurrence of PD sensitively, evaluate the peak value of the discharge inception voltage with random waveform and the damage extent relatively cost effectively. The interferograms affected by the PD are collected online. By extracting the phase information of the interference fringes quantitatively, the peak value of the discharge inception voltage with random waveform can be retrieved real-time. Merits of the proposed method as an online quantitative PD monitor are validated via theoretical analysis as well as experimentations by the use of an artificially localized PD source. Furthermore, the proposed method can capture the light signal emitted by the discharge. Quite in contrast to many commonly used sensor-based methods, our approach avoids the need of amplifying the light signal strength making its practical implantation much convenient. The proposed method promises strong potential for field application.

## Introduction

Partial discharge (PD) is a complicated physical phenomenon that occurs in high-voltage electrical equipment as a result of local enhancement of the electric field. Effects of slight PD on the insulation of power equipment may be minor in the short run. However, strong PD will cause the insulation strength to drop rapidly causing serious insulation damage in high-voltage electrical equipment^1^. PD is not only the main cause of insulation deterioration but also a key symptom and manifestation of insulation deterioration. To this end, the PD detection and analysis has been much investigated research topic by research institutions, equipment manufacturers, and power system operation departments^2,3^.

PD research and field application have made great progress also as an important evaluation method of electrical equipment insulation performance. As a result, the traditional preventive tests have gradually been replaced by online monitoring-based methods. Widely used traditional methods include pulse current method, ultra-high frequency (UHF) detection method, and ultrasonic detection method^4^. Pulse current method, which is the only internationally standardized method at present, has been widely used in cables, transformers, switchgears and the factory test of electrical equipment. This method is based on the pulse current signals caused by PD^5^. UHF detection method is based on the spectral characteristics of electromagnetic waves radiated by PD. Different detection bands have different sensitivity, anti-interference ability, pulse resolution and quantity of obtained information^6^. UHF based methods have mostly been used in the PD detection of transformers and gas insulation switchgears^7,8^. Electromagnetic signal attenuation due to refraction and reflection during the wave propagation reduces the detection accuracy, which makes detection difficult^9^. Ultrasound detection method has mostly been used in localization of the PD source^10^. Such methods adopt dielectric ultrasonic waves formed by instantaneous change of dielectric density caused by PD. At present, use of optical fibers for ultrasonic signal detection has shown promise^11^.

Most of the current researches and novel ideas are based on the combination of the traditional methods to complement each other for improved PD detection^12^. Tang combined pulsed current method and UHF method^13^; Huang and Zhou both combined ultrasonic and UHF detection methods^14,15^; Zhang combined the transient ground voltage, ultrasonic, and UHF detection methods^16^; Yoshida pointed out a more appropriate and advanced use of the UHF method^17^; Chen and Yang proposed to extract the main PD ultrasonic signal characteristics from the marginal and Hilbert spectra contributing to the diagnosis of PD faults^18^; Zhang analyzed the PD ultrasonic signals based on similar matrix blind source separation and convolution neural network^19^; Zhang and Si performed PD ultrasonic signal detection using optical fiber^20,21^. All the above researches have focused on combination and/or improvement of traditional methods at the cost of increased workload, operational difficulty, and cost.

There also are many other methods in rapid development such as chemical detection methods, photo detection methods and so on. Granado proposed two techniques allowing broadband PLC receivers to detect PD at the same time, but no experimental validation has been done^22^; Danouj highlighted the application potential of a new generation of piezoelectric sensors^23^; The system proposed by Shang used a Fabry–Perot interferometric sensor fabricated by photolithography^24^. A comprehensive review on sensor-based PD detection methods by Yaacob concluded that optical detection provides many advantages in terms of accuracy and suitability compared to other techniques^25^. Furthermore, implantation related issues in sensor-based methods have become real bottlenecks that limit development.

Considering the shortcomings of the traditional PD detection methods, their combination and improvement difficulties, sensor sensitivity and implantation related issues, and the cost, this paper proposes a novel cost effective setup for quantitative online PD monitoring. The setup only uses interferometry to achieve the aim of perceiving the occurrence of PD sensitively, evaluating the peak value of the discharge inception voltage to assess the damage extent, and providing important reference for the insulation state of the electrical equipment. With promising and realistic application potential, the setup can also perform online monitoring on the apparent discharge problems and evaluate the peak value of the discharge inception voltage with random waveform. Furthermore, it can also capture the light signal emitted by the discharge, which contributes to avoiding overvoltage interference and improving the detection credibility. Feasibility is verified both theoretically and experimentally.

## Theoretical analysis

To perceive the occurrence of PD sensitively and evaluate the peak value of the discharge inception voltage with random waveform, this paper proposes a quantitative interferometric monitoring setup. Firstly, its feasibility is verified in theory^26^.

The refractive index, *n*, is defined as the ratio of the propagation velocity of light in the vacuum, *c*, to the propagation velocity in the medium, *v*. Hence, *n* = *c*/*v* = (*ɛ*_*r*_*μ*_*r*_)^1/*2*^ where *ɛ*_*r*_ and *μ*_*r*_ are relative permittivity and relative permeability, respectively. Refractive index of the medium will change with the change of the permittivity and permeability.

When two coherent beams interfere, the combined light intensity distribution at any point in the interference field is:

1 $$ I = I_{R} + I_{S} + 2\sqrt {I_{R} I_{S} } \cos \frac{2\pi }{\lambda }\Delta $$

where *I*_*R*_ and *I*_*S*_ are light intensity of reference light and sample optical light, respectively, ∆ is the optical path difference between two beams reaching the same point, and λ is the wavelength.

The parameters of the reference optical path of the interferometer are kept unchanged and the tested system is placed into the sample optical path. When PD occurs, the electric field reflecting the peak value of the discharge inception voltage with random waveform will be generated around the sample optical path of the interferometer. According to the above theoretical knowledge, the refractive index of the medium in the sample optical path will be changed. Since the parameters of the reference optical path remain unchanged, the PD occurring in the sample path is equivalent to introducing an additional phase difference. At this point, the interference fringes formed by the superposition of two coherent laser beams will be offset and deflected.

The function, *c*(*x*, *y*), is obtained by performing Fast Fourier Transform (FFT) on the interferogram after filtering out the background light and other redundant information and only extracting the phase information in the positive first order spectrum, which is processed by inverse FFT. The phase distribution function with the information of the measured object is as follows:

2 $$ h_{0} (x,y) = \arctan \frac{{{\text{Im}} [c(x,y)]}}{{{\text{Re}} [c(x,y)]}} $$

where Re[*c*(*x*, *y*)] and Im[*c*(*x*, *y*)] are the real part and imaginary part of *c*(*x*, *y*), respectively. The phase principal values extracted by the arctangent are distributed between − π and + π. The phase distribution of the recovered wave-front is discontinuous. To eliminate the jump, the wave-front must be phase unwrapped to recover the true phase from the principal value or phase difference, which reflects the intensity of the electric field generated in sample optical path^27^.

To sum up, when PD occurs in the sample optical path of the interferometer, the electric field reflecting the peak value of the discharge inception voltage with random waveform will be generated, the refractive index of the medium in the sample optical path will change, which is equivalent to introducing additional phase difference, and the interference fringes will be offset and deflected. Furthermore, the higher the peak value of the discharge inception voltage, the stronger the intensity of the electric field introduced in the sample optical path. The greater the variation of the refractive index of the medium, the greater the additional phase difference^28^. In a word, the intensity of electric field is positively correlated with the peak value of the discharge inception voltage. The additional phase difference and the deformation degree of interference fringes are both positively correlated with the intensity of the electric field. By taking the intensity of the electric field as the intermediate quantity, the relationship between the phase difference and the peak value of the discharge inception voltage is established. Therefore, the peak value of the discharge inception voltage can be retrieved by the recovery of the phase difference, and the PD can be detected online.

## Experiment

**Figure 1:**
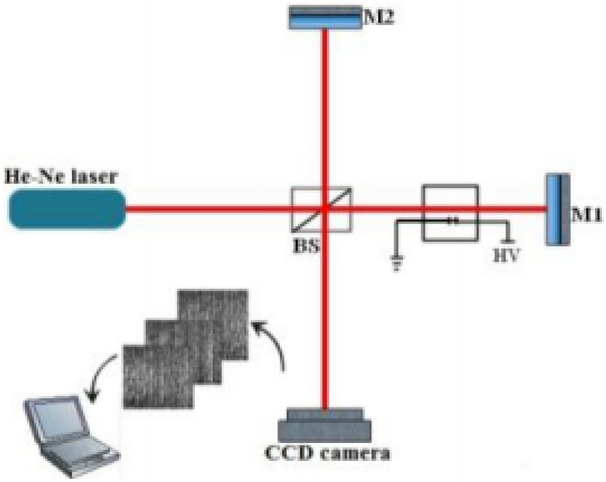
Experimental setup.

Based on the theoretical background presented in the preceding section, an experimental platform was built with an artificially localized PD source to further verify the feasibility as shown in Fig. 1. The experimental setup included an interferometer, CCD camera, and a localized PD source. A Michelson interferometer, consisting of He–Ne laser, beam splitter (BS), and two reflector mirrors (M1 and M2), was set up to detect PD of the localized PD source.

The wavelength of He–Ne laser was 632.8 nm. The laser beam could be separated into two beams with equal intensity via beam splitter (BS), the sample optical path, and the reference optical path. Without any additional condition, there would be no difference between the sample optical path and reference optical path. The interference fringes formed by the interferometer were projected onto the CCD camera (Sun Time 200A, China, with 640 × 480 number of 3.2 μm pixels). After photoelectric conversion, the CCD camera transmitted the received interferograms to the computer enabling real-time reproduction of the interferograms on the computer screen. As a result, the interferograms could be collected online.

**Figure 2:**
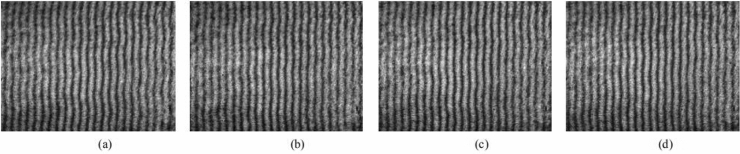
(**a**)–(**d**) interferograms effected by PD of 5 kV occurring above the sample optical path 2 cm, 1 cm, and below it 1 cm, 2 cm, respectively.

**Figure 3:**
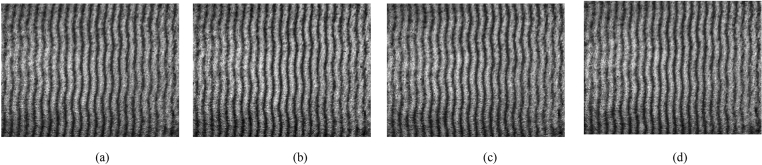
(**a**)–(**d**) interferograms effected by PD of 5.5 kV occurring above the sample optical path 2 cm, 1 cm, and below it 1 cm, 2 cm, respectively.

**Figure 4:**
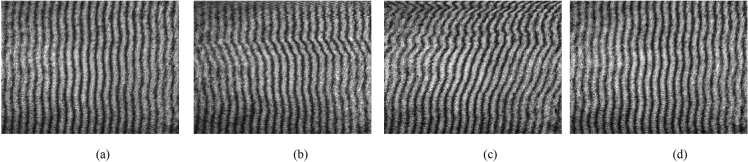
(**a**)–(**d**) interferograms effected by PD of 6 kV occurring above the sample optical path 2 cm, 1 cm, and below it 1 cm, 2 cm, respectively.

**Figure 5:**
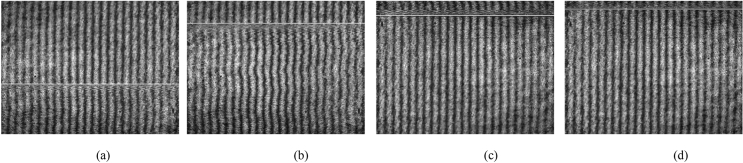
(**a**)–(**d**) interferograms with the light signal emitted by PD of 6 kV occurring above the sample optical path 2 cm, 1 cm, and below it 1 cm, 2 cm, respectively.

This way, PD occurring around the sample optical path could be detected and recorded. The proposed method could also retrieve the peak value of discharge inception voltage with random waveform. In the experiment, the artificially localized PD source was generated by the use of a lightning surge generator (SUG61005TB, China) and two identical electrodes. Lightning surge generator was as pulse voltage source. The length of the electrodes was 1.5 cm and the distance between them was 0.5 cm. One of the electrodes was connected to HV and the other to the earth potential. The continuous pulse output interval of the lightning surge generator was 60 s, the waveform was 1.2/50 μs, and the peak values were 6 kV, 5.5 kV and 5 kV, respectively. The artificially localized PD source was placed 1 cm below and 2 cm above the sample optical path. When the interferograms formed without any additional condition appeared at the computer screen, the lightning surge generator output the pulse. With high sensitivity, the interference fringes would be offset and deflected at the occurrence of PD. The interferograms captured online are shown in Figs. 2, 3, 4 and 5.

Single and continuous pulse experiments with different peak values were carried out 1 cm below and 2 cm above the sample optical path. As can be seen in Fig. 3, with the same inception discharge voltage, the deformation extent of the interference fringes increases as the distance between the PD source and sample optical path decreases; with the same distance, the deformation extent of the interference fringes increases as the voltage increases. These results are completely consistent with the theory. In addition, the light signal emitted by PD could be captured by the proposed method as shown in Fig. 5. Compared to the optical sensors used to detect PD, which usually rely on other technologies to amplify the light signal strength in addition to the implantation related difficulties, the proposed method shows its relative advantages in field applications.

## Result analysis

**Figure 6:**
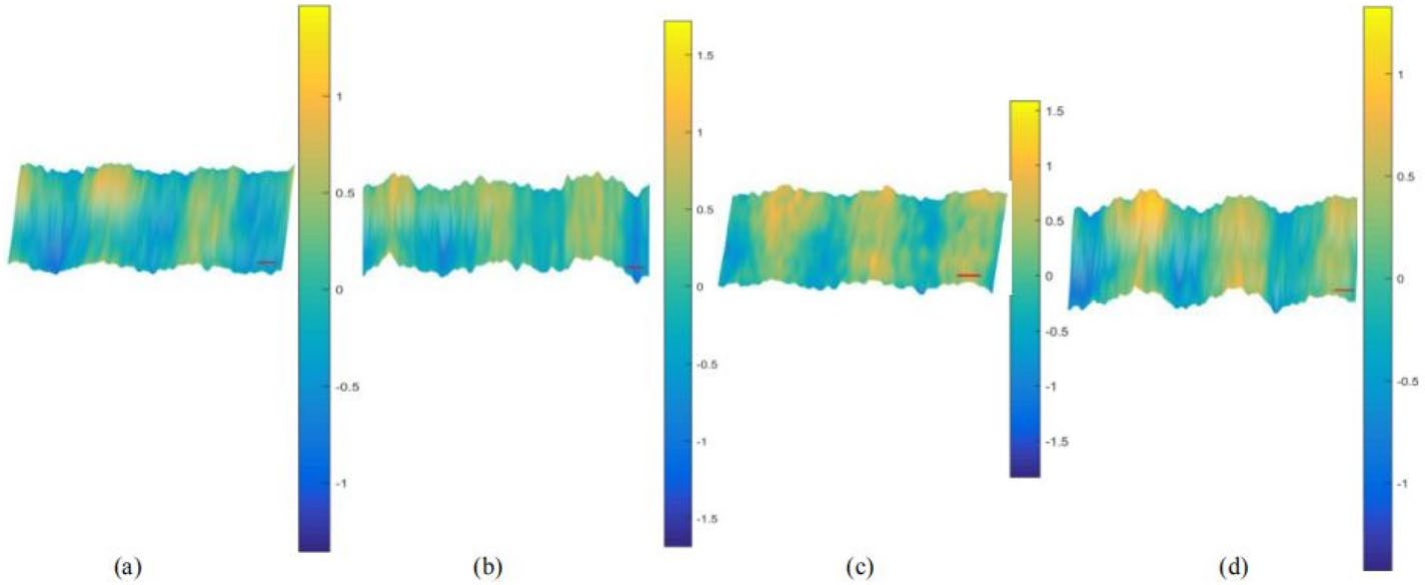
(**a**)–(**d**) phase distribution maps effected by PD of 5 kV occurring above the sample optical path 2 cm, 1 cm, and below it 1 cm, 2 cm, respectively.

**Figure 7:**
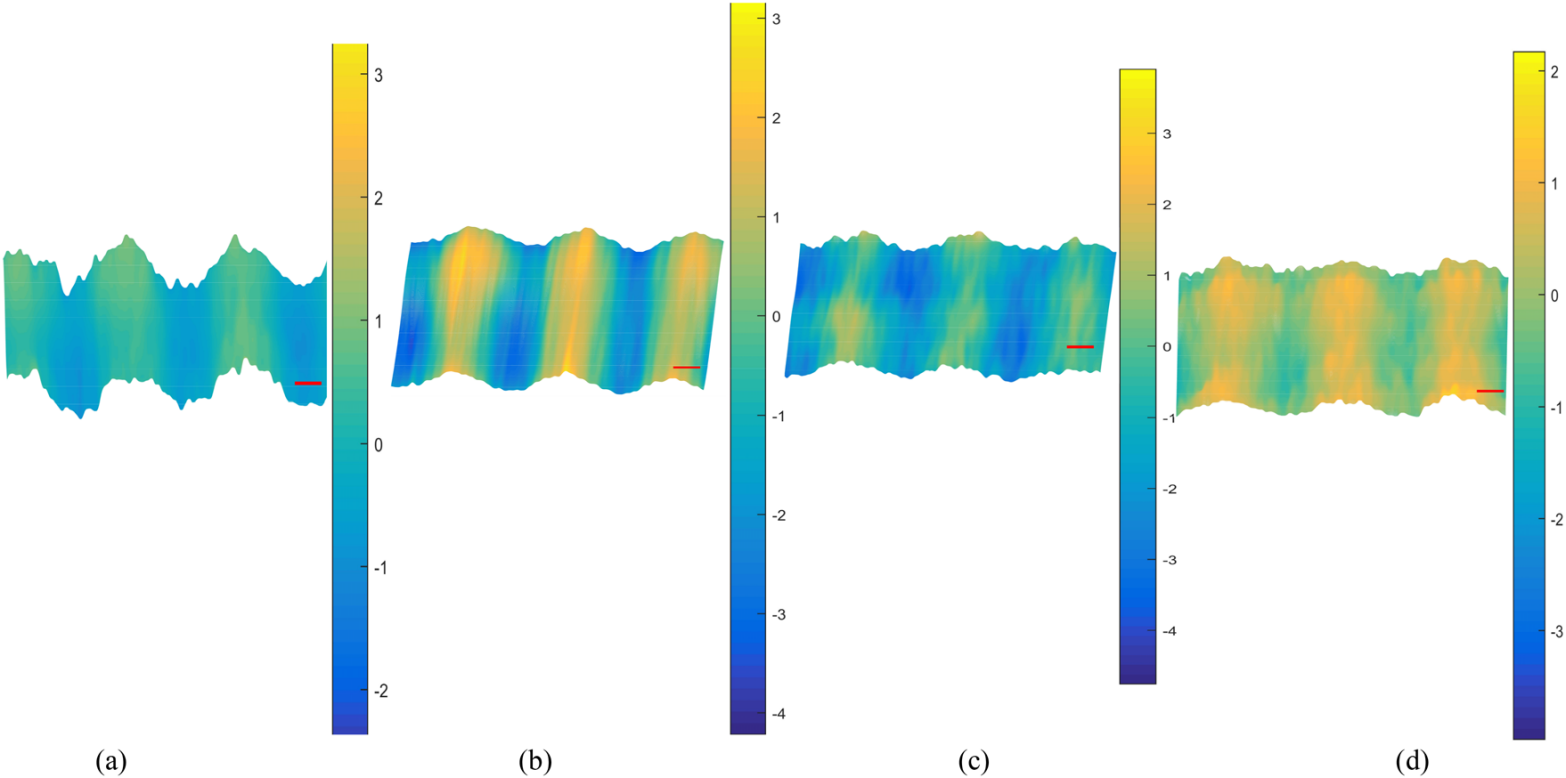
(**a**)–(**d**) phase distribution maps effected by PD of 5 kV occurring above the sample optical path 2 cm, 1 cm, and below it 1 cm, 2 cm, respectively.

**Figure 8:**
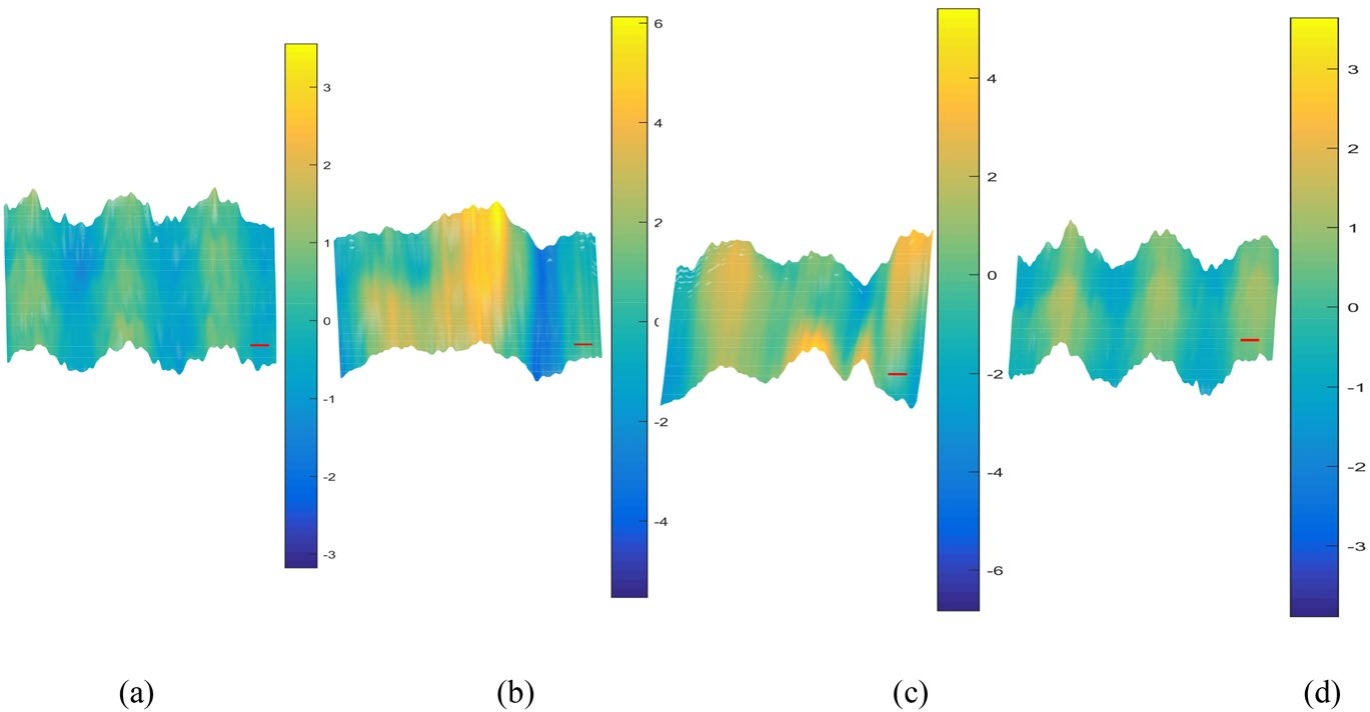
(**a**)–(**d**) phase distribution maps effected by PD of 5 kV occurring above the sample optical path 2 cm, 1 cm, and below it 1 cm, 2 cm, respectively.

**Figure 9:**
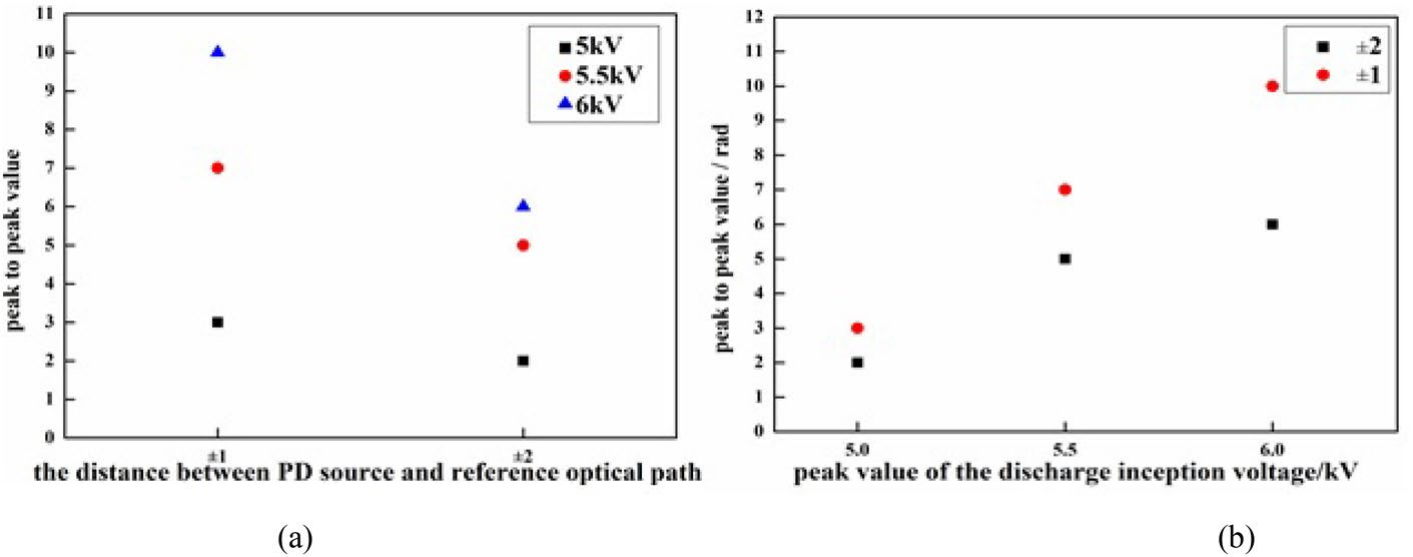
(**a**), (**b**) show the relationship between peak-to-peak value of phase distribution maps and the PD position, the peak value of the inception discharge voltage.

The phase information was extracted to obtain the phase distribution maps. The positive first-order spectrum containing phase information was extracted by performing FFT on the interferograms in a digital computer. Volkov unwrap processing was then carried out. Then, a smooth gradient was subtracted from the phase profile, which was smoothened with a 2D cubic spline smoothing filter before removing the hot pixels. Each step can be completed in seconds. Finally, the phase distribution maps containing the phase information of the above interferograms were obtained in real time as follows in Figs. 6, 7, 8 and 9.

Scatters were drawn to analyze the phase distribution law clearly. The relationship between peak-to-peak value of phase distribution maps and the PD position, the peak value of the inception discharge voltage can more intuitively be observed. When the peak value of the inception discharge voltage is constant, the closer the distance between the PD source and sample optical path, the larger the peak-to-peak value of phase distribution maps; when the distance between the PD source and sample optical path is constant, the higher the peak value of the inception discharge voltage, the larger the peak-to-peak value of phase distribution maps. As can be seen in Fig. 9, variation trend of the peak-to-peak value of phase distribution maps agrees well with the deformation extent of the interference fringes and theory. Intensity of the electric field is positively correlated with the peak value of the discharge inception voltage. Additional phase difference and the deformation degree of interference fringes are both positively correlated with the intensity of electric field. By taking the intensity of electric field as the intermediate quantity, the relationship between the phase difference and the peak value of the discharge inception voltage is established. As a result, the peak value of the discharge inception voltage could be retrieved by the recovery of the phase difference, and the PD can be detected online.

Besides, the light signal emitted by PD could be captured by the proposed method, avoiding the over-voltage interference and improving the detection credibility; The direction of light propagation of interferometer can be adjusted. With the equal optical distance between the sample optical path and reference optical path, the reflector can be introduced to change the propagation direction of the laser to bypass some obstacles; the proposed method can detect the PD occurring below and above the sample optical path, contributing to field test.

In addition to universality, sensitivity, cost effectiveness, and small volume, the proposed setup can withstand extremely high peak value of the inception discharge voltages. The setup can also perform on-line monitoring on the apparent discharge and evaluate the peak value of the discharge inception voltage with random waveform to provide a quantitative measure for performance analysis and protection of electrical equipment.

## Conclusion

By extracting the phase information of the offset and deflected interference fringes affected by PD online, the proposed method monitors the PD occurrence and retrieves the peak value of the inception discharge voltage with random waveform in real time. It has been intensively tested by artificially localized PD with the peak values of 5 kV, 5.5 kV and 6 kV occurring 2 cm above and 1 cm below the optical path to verify its feasibility. Moreover, the proposed setup can withstand extremely high peak value of inception discharge voltage. High sensitivity, low cost, less volume and workload, and its ability to detect the PD occurring at different relative positions make it suitable for field applications. It can also capture the light signal emitted by PD. This avoids the overvoltage interference and improves the detection credibility. The proposed method also shows its advantages in field application compared to optical sensors used to detect PD which usually rely on other technologies to amplify the light signal strength amidst the implantation difficulties.
